# Supplementary material for: YAP inhibits NF-κB signaling and ccRCC growth by opposing p65-ZHX2 cooperativity
Source: bioRxiv. 2024 Jun 27:2024.06.21.600079. Preprint. [Version 1] doi: 10.1101/2024.06.21.600079 (PMC11230290; doi:10.1101/2024.06.21.600079)
Supplement: Supplementary file 1 [file NIHPP2024.06.21.600079v1-supplement-1.pdf]

bioRxiv preprint doi: https://doi.org/10.1101/2024.06.21.600079; this version posted June 27, 2024. The copyright holder for this preprint (which was not certified by peer review) is the author/funder, who has granted bioRxiv a license to display the preprint in perpetuity. It is made available under aCC-BY 4.0 International license.

Xu_FigS1

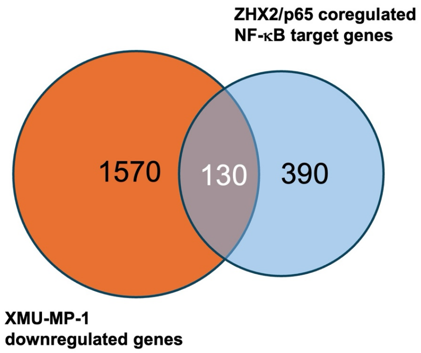

SCD	INSIG1	ABCC2	ABCC6	DSCAML1	VCAM1
CSF1	MEGF6	CARD6	IFIH1	IFI44L	CCL2
IRF9	C1orf106	HLX	MCTP1	RASSF9	VAV3
ABCA1	PTGER2	KY	NEFL	OLFML2B	CLEC4E
ZBTB38	CTHRC1	GRB7	TNFAIP3	ZHX2	CXCL8
SEC14L6	EPHB2	NID2	FOXP2	FRMD4B	C3
ZNF618	IFI44	BCL3	ARSE	IL1R1	KIF12
STEAP3	PER3	MAF	B3GNT4	CD24	TINAG
XAF1	ABLIM1	TLL1	TRIM22	STAT1	ITGB6
HGD	MRAS	MAML2	CNR1	SEMA3A	ENPP3
STAT6	MGLL	MSC	F8	HNF1B	ABCA12
C1QTNF6	IKBKE	DMRTA1	ACTR3C	FRK	SYNPO
PDZK1IP1	TNS1	TM4SF18	SLN	ARL14EPL	RNASET2
DDX60	LXN	SORCS2	OAS2	C1orf186	TMEM141
AHNAK2	ARHGEF6	TLR3	MMP1	CYP1B1	SEMA6A
PORCN	GCNT1	QPRT	FBXL13	SGPP2	SEMA5B
TNFSF10	DDX60L	SGK2	SLC44A3	OAS1	AGMO
TRIM38	BRINP1	PDGFB	PRKCQ	EFHB	TRABD2B
TNIP1	RYR3	SLC4A4	MAP7D2	SAA1	NCKAp5
RBPMS	MFNG	LIPG	GBP2	PLEK	TNFAIP6
ADAMTS9	IL6	PTGES	MTSS1	C14orf105	
TNS3	SHROOM4	MITF	SAMD9L	GPRC5B	

Fig. S1. XMU-MP-1 inhibits NF-κB target genes co-regulated by p65 and ZHX2

Overlap of XMU-MP1 downregulated genes and NF-kB target genes co-regulated by ZHX2 and p65 in 786-O cells (left). List of the 130 NF-kB target genes co-regulated by ZHX2 and p65 and downregulated by XMU-MP1 in 786-O cells (right).

bioRxiv preprint doi: https://doi.org/10.1101/2024.06.21.600079; this version posted June 27, 2024. The copyright holder for this preprint (which was not certified by peer review) is the author/funder, who has granted bioRxiv a license to display the preprint in perpetuity. It is made available under aCC-BY 4.0 International license.

Xu_FigS2

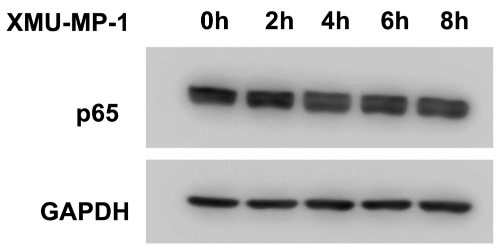

Fig. S2. Hippo pathway inhibition does not affect p65 protein level

Western blot analysis of p65 protein expression in 786-O cells treated with 2 μM XMU-MP-1 for the indicated time. GAPDH was used as a loading control.

bioRxiv preprint doi: https://doi.org/10.1101/2024.06.21.600079; this version posted June 27, 2024. The copyright holder for this preprint (which was not certified by peer review) is the author/funder, who has granted bioRxiv a license to display the preprint in perpetuity. It is made available under aCC-BY 4.0 International license.

Xu_FigS3

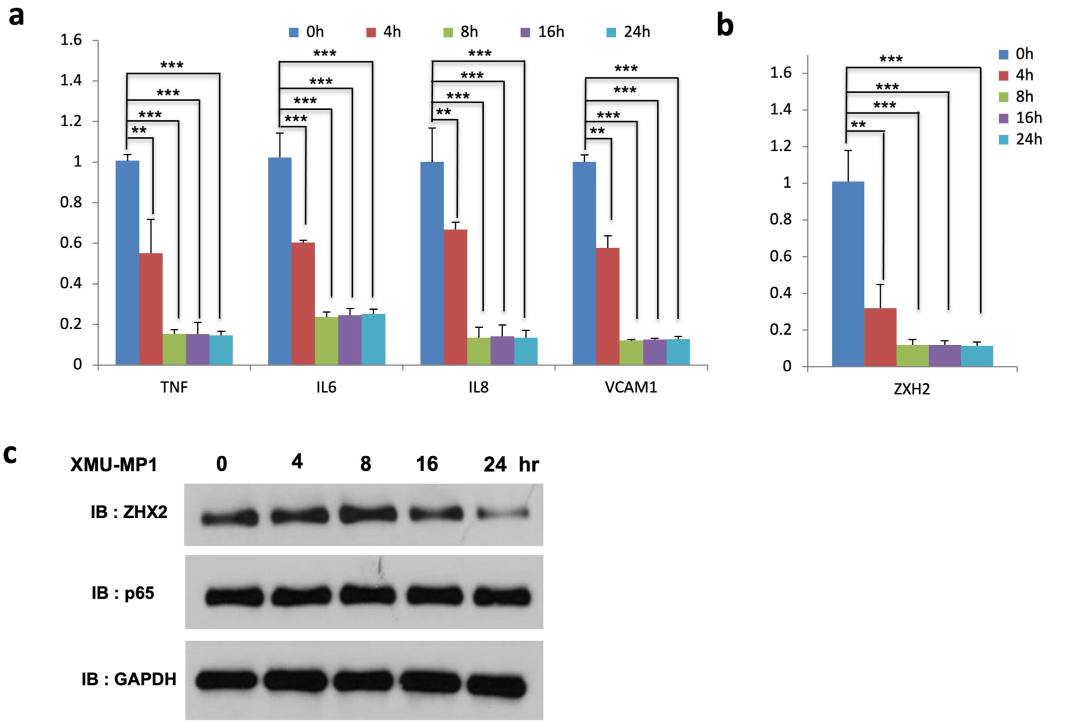

Fig. S3. XMU-MP-1 inhibits NF-κB target gene expression without affecting ZHX2 protein level

a, **b** Relative mRNA levels of the indicated NF-κB target genes (**a**) or *ZHX2* (**b**) in 786-O cells treated with 2 μM XMU-MP-1 for the indicated periods of time. Data in are ± SD. **P<0.01, ***P<0.001 (Student's t-test).

c Western blot analysis of ZHX2 and p65 protein expression in 786-O cells treated with 2 μM XMU-MP-1 for the indicated periods of time. GAPDH was used as a loading control. ZHX2 level started to decline after XMU-MP-1 treatment for 16 hours while p65 protein level remained unchanged even after 24 hours' treatment.
